# Inconsistencies and questionable reliability of the publication “Immunotherapy of metastatic colorectal cancer with vitamin D-binding protein-derived macrophages-activating, GcMAF” by Yamamoto et al

**DOI:** 10.1007/s00262-014-1587-y

**Published:** 2014-07-24

**Authors:** Ana Ugarte, Gauthier Bouche, Lydie Meheus

**Affiliations:** The Anticancer Fund, Boechoutlaan 221, 1853 Strombeek-Bever, Belgium

**Keywords:** Breast Cancer, Colorectal Cancer, Metastatic Colorectal Cancer, Colorectal Cancer Patient, Viral Component

Dear Editors,

After several patients asked our organization, the Anticancer Fund, www.anticancerfund.org, about GcMAF as a cancer treatment, we looked for the evidence supporting its use in cancer. The literature showed us striking issues and inconsistencies. We would like to comment on the article from Yamamoto et al. published in your journal in 2008 [1].

It is claimed that eight colorectal cancer patients were successfully treated with GcMAF, a protein claimed to be discovered by the authors. “Treatment success” was determined by Nagalase in serum. Nagalase is supposed to deglycosilate naturally occurring GcMAF in cancer patients so that it is incapable of activating macrophages to fight cancer. GcMAF manufactured by Yamamoto might be unaffected by Nagalase.

This article was published in parallel to two other articles by the same group in other journals, claiming that their product (GcMAF) successfully treated prostate [2] and breast cancer [3]. In 2009, they published another article reporting that GcMAF successfully treated HIV, again determining success with Nagalase [4]. In the cancer-related articles, the authors claim Nagalase is exclusively produced by cancer cells as opposed to the HIV-related article where they claim Nagalase is a viral component.

The authors do not give the most basic information on the disease of these patients: No TNM, no stage, no histology. They determined that these patients had metastatic disease, based exclusively on an elevated level of serum Nagalase. Nagalase is not a criterion to define metastatic disease in the TNM classification of cancer [5]. No key opinion leader has validated its use in oncology.

The claim *“Although their serum Nagalase activities indicated that they have significant amounts of metastasized tumor cells, CT did not detect metastasized tumor lesions in other organs”* means these patients did not have residual disease before starting GcMAF. All evidence cited to justify Nagalase use in this trial is publications by the same group (33 references that are cited 155 times against the 18 times the remaining 15 references are).

We have found the following about Yamamoto’s work:

1. The Nagasaki and the Hyogo Immunotherapy Research Groups, that gave IRB approval for these trials, do not exist except in Yamamoto’s clinical papers. Three purported members of these groups, including one chairman, informed us they are not part of these groups and that they have never been involved in Yamamoto’s activities. Other members of these IRBs could not be found.
2. Yamamoto’s co-authors in these papers could not be found.
3. We contacted the sponsors of these trials (US Public Health Service and the Elsa U. Pardee Foundation), and we found that they did not support them. They only supported Yamamoto’s early preclinical work while he was affiliated to other institutions rather than his Socrates Institute for Therapeutic Immunology.

This article also contains many mistakes and uses invalid endpoints:

1. Many references are used inappropriately and most do not support the authors’ claims. For example: The assertion *“Administration of 100 nanogram (ng) GcMAF to humans results in the maximal activation of macrophages with 30*-*fold increased ingestion index and 15*-*fold increased superoxide*-*generating capacity”* has no basis. This statement is supported by reference 33, which is an animal experiment in which these numbers are not mentioned. Furthermore, it has been demonstrated that naturally occurring GcMAF in cancer patients has a concentration of approximately 4 mg/L, making the 100 ng proposed by Yamamoto meaningless, plus it is not deglycosilated [6].
2. Without adequate randomized controlled clinical trials, the assertion *“Since the molecular structure of GcMAF is identical to that of the native human MAF, GcMAF (even 5*-*fold higher therapeutic dosage) produced no side effects”* is wrong and dangerous. It is well established that injection of some human products (i.e., insulin and epinephrine) into patients can be lethal.
3. The conclusions make no sense: *“The curative rate measurements of tumors during GcMAF therapy and the estimation of the degree of tumor differentiation have been possible because of the availability of precision measurement of serum Nagalase”*. Yamamoto proved that Nagalase failed as a disease measurement method when it was compared to CT scans at the beginning of the study. However, at the end of the study, when the CT scans matched the authors’ speculations, CT scans were again reported. The degree of tumor differentiation can only be determined by histopathology, which was not reported in this or their other articles (prostate and breast cancer articles).

These results cannot be scientifically validated as they contradict established tenets in oncology.

## Abbreviations

CT: Computerized tomography
GcMAF: Gc protein-derived macrophage-activating factor
HIV: Human Immunodeficiency Virus
IRB: Institutional Review Board
MAF: Macrophage-activating factor
